# Microplastics Distribution within Western Arctic Seawater and Sea Ice

**DOI:** 10.3390/toxics11090792

**Published:** 2023-09-20

**Authors:** Alessandra D’Angelo, Nicole Trenholm, Brice Loose, Laura Glastra, Jacob Strock, Jongsun Kim

**Affiliations:** 1 Graduate School of Oceanography, University of Rhode Island, Narragansett, RI 02882, USA; 2 Center for Environmental Science, University of Maryland, Cambridge, MD 21613, USA; ntrenholm@umces.edu; 3 School of Earth, Environmental and Marine Sciences, The University of Texas Rio Grande Valley, Brownsville, TX 78520, USA

**Keywords:** microplastics, marine litter, sea ice, Canadian Arctic Archipelago, Arctic marginal seas

## Abstract

Microplastic pollution has emerged as a global environmental concern, exhibiting wide distribution within marine ecosystems, including the Arctic Ocean. Limited Arctic microplastic data exist from beached plastics, seabed sediments, floating plastics, and sea ice. However, no studies have examined microplastics in the sea ice of the Canadian Arctic Archipelago and Tallurutiup Imanga National Marine Conservation Area, and few have explored Arctic marginal seas’ water column. The majority of the microplastic data originates from the Eurasian Arctic, with limited data available from other regions of the Arctic Ocean. This study presents data from two distinct campaigns in the Canadian Arctic Archipelago and Western Arctic marginal seas in 2019 and 2020. These campaigns involved sampling from different regions and matrices, making direct comparisons inappropriate. The study’s primary objective is to provide insights into the spatial and vertical distribution of microplastics. The results reveal elevated microplastic concentrations within the upper 50 m of the water column and significant accumulation in the sea ice, providing evidence to support the designation of sea ice as a microplastic sink. Surface seawater exhibits a gradient of microplastic counts, decreasing from the Chukchi Sea towards the Beaufort Sea. Polyvinyl chloride polymer (~60%) dominated microplastic composition in both sea ice and seawater. This study highlights the need for further investigations in this region to enhance our understanding of microplastic sources, distribution, and transport.

## 1. Introduction

Microplastics (MPs) are the predominant type of marine litter, as recorded by GESAMP in 2010 [1] and the LITTERBASE website [2]. These particles consist of a polymer-based structure and vary in size from 1 μm to 5 mm [3]. Secondary MPs are generated through the degradation of larger plastic objects. The deterioration of MPs is influenced by various factors, including pH, salinity, temperature, UV exposure, biofouling, and physical fragmentation, as demonstrated in previous studies [4,5]. Notably, the examination of MPs in the Arctic region has primarily focused on sampling from marine surface layers (e.g., [6]) and the seafloor (e.g., [7]), as documented by the LITTERBASE website [2] (https://litterbase.awi.de/, accessed on 10 July 2023) and a recent review [8]. Several investigations have been conducted on MPs in Arctic sea ice, and a few studies have been conducted along the Alaskan shelf, in the Chukchi and Beaufort seawaters (e.g., [6,9,10]). Due to the limited number of studies and restricted extent of global observation, there is still a limited representation of MP concentrations worldwide.

Microplastics have been found in the sediment, sea surface, and sea ice of the Arctic Ocean [6,7,11,12,13,14] and are likely to come from ocean currents of Pacific and Atlantic origin as well as from atmospheric deposition [15,16]. Long-distance transport to remote regions could occur through a combination of atmospheric and marine conveyance [17,18], facilitating the global spread of MPs. Recent studies highlighted the occurrence of MPs in atmospheric aerosol and snow for example [16,19]. Estimates of plastic entering the ocean vary annually between 4.8 to 12.7 million metric tons, while floating marine plastic is calculated to be only 268,940 tons, accounting for just 2–6% of the estimated plastic entering marine systems every year [20].

As a result, the transport processes that govern the fate and ecological impact of microplastics remain largely unknown. Consequently, it is essential to investigate the distribution and deposition of microplastics in environmental compartments to gain a deeper understanding of these transport mechanisms and develop predictive models for identifying hotspots, assessing ecological risks, and implementing mitigation strategies.

Here, we present the MP data obtained from two Arctic campaigns: the Northwest Passage Project, which took place in July and August 2019 across the Canadian Arctic Archipelago (CAA) aboard the Swedish RVIB Oden, and the SKQ0202014S Cruise, conducted in October and November 2020 on the United States RV Sikuliaq. These two campaigns involved the collection of samples from distinct regions and matrices, and therefore, they are not intended for direct comparison. Our study aims to provide valuable microplastics data from understudied regions where existing data are limited. While born from opportunistic samplings, our research was developed with the precise goal of addressing critical knowledge gaps in understanding microplastic distribution and accumulation in these specific locations. By analyzing carefully selected samples from both sea ice and seawater environments, we seek to explore potential differences in microplastic dynamics and uncover unique insights into pollution sources and environmental characteristics. Through our study, we aim to contribute scientifically relevant and meaningful data to address the broader issue of microplastic pollution in regions where information is scarce. As highlighted in recent literature, e.g., [21,22,23,24,25], MPs have been observed to enter the marine food web and, through the process of bioaccumulation, pose potential harm to organisms. During the NSF-funded cruise in the CAA, we examined five sea ice cores to determine microplastic concentrations, identify different types of microplastics, and study their vertical distribution within the sea ice. Additionally, during the SKQ0202014S Cruise, we collected 17 water samples from specific depths in various locations along the Chukchi and Beaufort Shelves to analyze the microplastic content within the water masses.

### Area of Study

**Figure 1:**
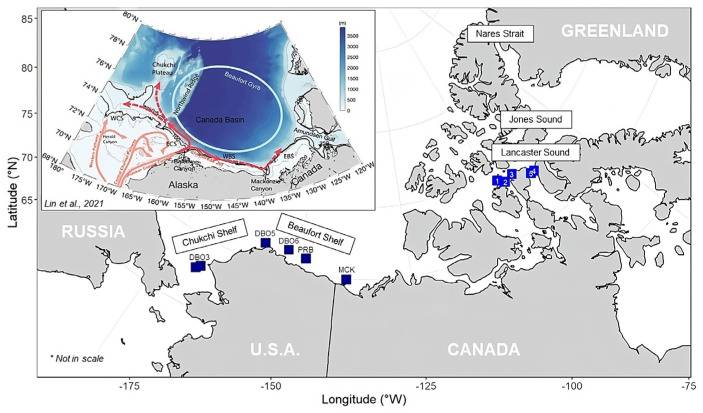
Map of the sampling stations, with overlapped schematic circulation of the western Arctic Ocean and place names. The main flow pathways of Pacific water across the Chukchi shelf are indicated by the light red arrows. The Barrow Canyon outflow, Beaufort Shelf break jet, and Chukchi Slope Current are marked by the red arrows, where the dashed portions indicate less certainty. The schematic Beaufort Gyre is marked by the light blue circle. WCS: western Chukchi Sea; ECS: eastern Chukchi Sea; WBS: western Beaufort Sea; EBS: eastern Beaufort Sea [26]. The dark blue squares show the SKQ0202014S sampling sites, with labels displaying the station ID (Distributed Biological Observatory—DBO, Mackenzie Sound—MCK, and Prudhoe Bay—PRB); the light blue squares represent the Northwest Passage Sea ice samples, with the label representing the sea ice core ID.

The study area encompassed the Chukchi and Beaufort Shelves as well as the Canadian Arctic Archipelago (CAA), as illustrated in Figure 1. In this region, Pacific water is transported eastward toward the Beaufort Sea. The primary route for this transport is northward through the Bering Strait, which flows through the Chukchi Sea and into the western Beaufort Sea [26,27]. Along the Alaskan coast, the predominant pathway for Pacific water is the Alaskan Coastal Current (see Figure 1), which flows from the Bering Sea northeast through the Chukchi Sea and into the Beaufort Sea. This current facilitates the seasonal movement of water from Barrow Canyon (Figure 1) both westward as the Chukchi Slope Current, and eastward into the Beaufort Sea as the Beaufort Shelf break jet [26,28,29,30]. Water at the surface that is not of Pacific origin can typically be attributed to the melting of sea ice and the inflow of river water [18,26,31]. The Canadian Arctic Archipelago (CAA) plays a significant role in the transport of freshwater between the Arctic Ocean and the Atlantic Ocean [32]. Specifically, Nares Strait, Jones Sound, and Lancaster Sound (Figure 1) are the main channels that capture most of the eastward outflow from the CAA [33].

The significant declines observed in multi-year ice (MYI) in the Canadian Arctic Archipelago (CAA) and the Beaufort Sea are relevant to the aforementioned water mass transports. As mentioned earlier, Pacific water is transported through the Bering Strait into the Chukchi Sea and further into the western Beaufort Sea. The loss of MYI in the Beaufort Sea, which has exceeded that of the north-facing coast of the CAA, indicates a reduction in the presence of thick sea ice along the pathway of Pacific water transport [34]. It is noteworthy that the north-facing coast of the CAA still maintains some of the thickest sea ice globally, demonstrating its resistance to melting compared to thinner ice in other regions. The research conducted by Howell and Brady (2019) [35] suggests an increase in ice area fluxes from the Arctic Ocean into the CAA. This increased ice exchange aligns with the movement of ice southeastward across the CAA during the melt season. This seasonal movement and transport of thick multi-year ice (MYI) from the Arctic Ocean basin into the CAA has been described in previous studies [36]. The prolonged period of open water, resulting from thinning sea ice, enables faster ice movement and contributes to the increased exchange of ice between the Arctic Ocean and the CAA from 1997 to 2018 [35]. Furthermore, the expansion of open water areas, driven by thinning sea ice, has provided more opportunity for ice transport into the CAA. This expansion is associated with a recent shift where the southern regions of the CAA experience a greater inflow of multi-year ice (MYI) [35]. The dominance of first-year ice (FYI) in the southern regions of the CAA has been a typical pattern, but the increase in MYI inflow indicates changes in ice dynamics and the potential influence of open water areas. Studies conducted by Howell et al. (2006, 2013) [37,38] support these observations of ice exchange and inflow patterns within the CAA.

## 2. Materials and Methods

All the information and data pertaining to the samples are presented in Tables S1–S3.

### 2.1. Samples Collection

#### 2.1.1. Sea Ice

During the Northwest Passage Project conducted in July and August 2019, a total of five sea ice cores were collected (Table S1) using a Kovacs ice coring system with a diameter of 9 cm. The sampling locations were accessed via helicopter, and the sampling sites were selected based on sea ice concentration and logistical considerations. The easternmost sample was obtained from Prince Regent Sound, where the sea ice remained relatively consistent during the cruise. Sea ice concentration data, with a spatial resolution of 1 km, were obtained from the University of Bremen data archive (seaice.uni-bremen.de [39,40]). Additionally, sea ice charts provided by the Canada Ice Centre (www.canada.ca/en/environment-climate-change/services/ice-forecasts-observations/publications/interpreting-charts, accessed on 1 September 2021) were utilized. The cores were drilled and collected into black plastic bags for transportation. The samples were rinsed with deionized water (DIW) once onboard and cut using a stainless steel saw into two sections at 20 cm to preserve vertical resolution. In conjunction with sea ice cores, we collected under-ice water samples (1 L) using hand bottles to observe the microplastic concentration beneath the sea ice. After cutting, the sea ice was rinsed again with DIW and stored in multilayer foil bags (capacity 3 L, Restek, Bellfonte, PA, USA) until it was melted for further analysis.

#### 2.1.2. Seawater

In October–November 2020, aboard the research vessel Sikuliaq, we collected a total of 17 water samples (see Table S2). These samples were obtained at 9 stations spanning from the Bering Strait to the Canadian waters of Mackenzie Sound. A total of 12 L of water was filtered for microplastics from each sample. We employed a 15-micron mesh sieve, which was directly attached to the Niskin bottle used for water collection. This filtration process allowed us to concentrate the microplastics present in the water, making them more accessible for subsequent analysis and quantification.

By filtering a substantial volume of water through the 15-micron mesh sieve at the point of collection, we aimed to maximize the chances of capturing microplastics and obtaining representative data for our study. This approach allowed us to assess the microplastic content accurately and gain valuable insights into their distribution and abundance in the sampled environment.

The collection of samples occurred during various transects conducted throughout the cruise. The average water depth for sampling was approximately 85 m, with the maximum depth reaching 500 m. The majority of the samples were acquired from Distributed Biological Observatory (DBO) stations, which were strategically selected for this study, as it marked the inaugural instance of microplastic collection/sampling during a DBO cruise. Specifically, we prioritized sampling water layers situated above a depth of 50 m, which accounted for 11 out of the 17 total samples collected.

### 2.2. Samples Processing

The derived particle numbers per liter (MP.L) of melted ice and per liter of seawater were calculated from the number of particles (n.MP) and the volume fraction (Volume.L) of the sample. Particle numbers obtained from blank samples (control) were subtracted from the particle count (Table S3). Figure S1 shows the calculated error for each sample and site.

(1) $$MP.L=\frac{\left(n.MP\right)-control}{Volume.L}$$

#### 2.2.1. Sea Ice

The sea ice samples were melted inside the foil bags, and upon complete melting, were pre-filtered through a 5-micron sieve and then filtered under vacuum onto glass microfiber (GF/F) filters (Whatman Grade GF/F, 0.7 µm pore size, 25 mm diameter circle) on a clean bench on board the vessel. The filtered samples were then stored in the dark at −20 °C for subsequent analysis after the cruise. Due to capacity constraints, we only performed subsetting of the core into horizons starting from core 3. Subsequently, we decided to establish a vertical resolution to assess the vertical distribution of microplastics (MP), which is a well-established approach utilized in previous studies.

Once on land, the filters were examined using a Zeiss stereoscope to count and characterize the plastic particles at the University of Rhode Island (URI) Graduate School of Oceanography (GSO). Furthermore, the filters were subjected to polymer composition analysis using a Raman WITec alpha 300 R microscope at the URI Rhode Island Consortium of Nanoscience and Nanotechnology (RIN2). A comprehensive description of the detailed procedure can be found in the supplementary material (SOPs S1, S2, S3). The sizes of the microplastics detected at the stereoscope and Raman microscope ranged from 10 µm to 1 mm. Particles smaller than 10 µm were not detected, likely leading to an underestimation of the concentration of microplastics in the sea ice cores.

#### 2.2.2. Seawater

The under-ice seawater collected during the NPP cruise was filtered using a 5-micron sieve and subsequently examined onboard using stereoscopy (see Table S3 for results). Water column samples obtained during the SKQ0202014S cruise were filtered using a 15-micron sieve directly from the Niskin bottles. These filtered samples were then preserved in bottles containing 5% formalin to prevent biologically driven degradation of microplastic particles until they could be analyzed post-cruise. Water samples were handled cautiously (see Section 2.3) to avoid introduction of microplastic contaminants. The protocols reported in the supplementary material (SOP S1 Steps 1–4, 7 and 8) and Miller et al. 2017 [41] were followed. The sizes of the microplastics detected at the stereoscope and Raman microscopes ranged from 10 µm to 1 mm. Particles smaller than 10 µm were not detected, likely leading to an underestimation of the concentration of microplastics in the seawater.

### 2.3. Contamination Control

To ensure accurate and reliable analysis of microplastics throughout the process, stringent quality control measures were implemented to prevent contamination. To exclude sources of synthetic polymers at each sampling stage, we ensured that personnel wore non-fibrous clothing. The lab had separate spaces for sample preparation, processing, and analysis. These areas were regularly cleaned and maintained to minimize the presence of microplastics and prevent cross-contamination. All glassware and other materials were thoroughly washed and rinsed with deionized water and 70% ethanol, to prevent particles from adhering to the surfaces.

The coring system was constructed using fiberglass with an aluminum head, whereas seawater samples were collected using plastic bottles (during NPP) and Niskin bottles (during SKQ0202014S).

To assess external contamination in plastic bottles, a blank sample was conducted during NPP. For this, one bottle was filled with deionized water, and the blank sample was processed alongside the real samples obtained from the environment. This procedure was performed once, considering resource and logistical constraints. The results of the blank sample showed no contamination. Although it would have been ideal to conduct the field blank concurrently with each sampling collection, the confidence in the reliability of the data was upheld due to the results obtained.

During SKQ0202014S our capacity was limited, and we could not perform field blanks. Following SKQ0202014S seawater collection, the samples were filtered using a 15-μm metal sieve, which had been pre-rinsed with deionized water and 70% ethanol. To minimize contamination risk, the sieve was covered with aluminum foil, and plastic particles were rinsed directly into clean bottles containing 5% formalin. The entire process was carried out on a clean bench to ensure further prevention of contamination.

Regular quality control checks such as running blank samples were conducted throughout the analysis process (Table S4). All data were corrected using the data from daily blank samples (see Equation (1)).

### 2.4. Raman Processing

Microplastics were analyzed using a WITec alpha300 R confocal Raman microscope equipped with a CCD camera, motorized XYZ stage, and 785 nm laser source. Spectra were acquired with the laser power set to 10 mW and an integration time of 1 s × 300 accumulations (Figures S2 and S3). The spectral processing was performed using the WITec Five software version 5.2 (SOP S2).

### 2.5. Software for Polymer Identification

Once the Raman spectra were acquired, they were translated into identifiable plastic types (see Figure S4) using the siMPle software version 1.1, which utilizes a library of reference spectra for plastics version 1 [42]. Briefly, the processing algorithm calculated a score by correlating the raw spectra, their first derivatives, and their second derivatives through Pearson correlation. This resulted in three Pearson’s correlation coefficients for each combination of the sample spectrum and reference spectrum. The squared R-values were then assigned global weights by the user. The algorithm generating the score correlated the raw spectra, their first derivatives, and their second derivatives via a Pearson correlation, yielding three Pearson’s correlation coefficients (r0, r1, r2) per combination of sample spectrum and reference spectrum. The r values were squared, and global weights (k0, k1, k2) were assigned by the user. A score between 0 and 1 were calculated as Sd(i,j), where (i,j) are the coordinates of the pixel on the map and d is the number of the reference spectrum (Equation (2)). Negative correlations were omitted, i.e., if an R-value was below zero, it was set to zero [43].

(2) $$Sd\left(i,j\right)=\frac{\left(k0r0^{2}+k1r1^{2}+k2r2^{2}\right)}{k1+k2+k3}$$

## 3. Results

### 3.1. Microplastics in Canadian Arctic Sea Ice

**Figure 2:**
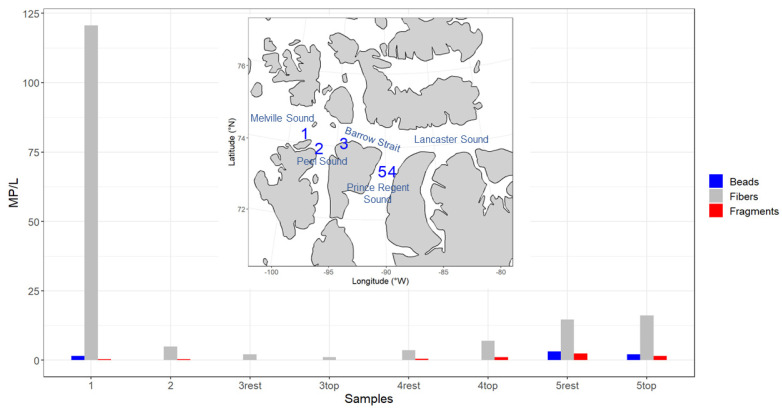
Microplastic concentration (MP/L) for each core, color-coded by shape type, and accompanied by a map indicating sample locations. Cores three, four, and five were subdivided into two distinct sections: the top section and the remaining portion of the core.

The sea ice samples obtained during the Northwest Passage Project exhibited an average salinity of 1.98 ± 1.17 (https://doi.pangaea.de/10.1594/PANGAEA.937543, accessed on 1 May 2023). These values suggest that the sea ice was most likely multi-year ice (MYI) that originated from the Central Arctic, based on previous studies [44,45,46,47]. Several factors, including gravity drainage, result in decreasing salinity of the ice as it ages; MYI has a much lower salt and air content than first-year ice due to the melting and refreezing processes it has experienced [48]. The average MP per liter occurring in the sea ice was 23 ± 11, which is much higher than the seawater average (1.9 MP/L). Microplastics were found to be most abundant in cores one and five, with a prevailing filament shape type observed (Figure 2). This indicates that there is no discernible spatial trend within the sites investigated in the study area.

**Figure 3:**
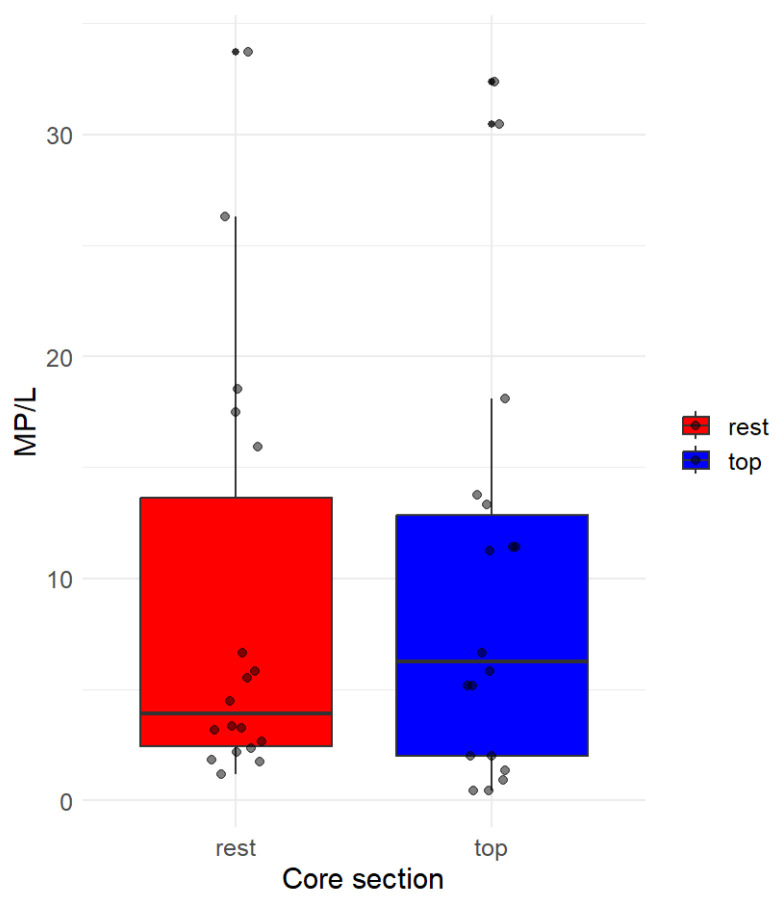
Box plot illustrating the distribution of MP/L across different core sections. Each box plot represents the variation in MP/L for the respective core section, with red and blue hues denoting the top and the rest of the cores. Jittered points are overlaid on the box plots to visualize individual data points within each group. The *x*-axis corresponds to the core sections, while the *y*-axis represents the MP/L values.

Concerning the analysis of the layers separately, we employed the Welch two sample t-test to investigate any potential significant differences in the MP concentration values between the two groups “top” and “rest” (Figure 3). The t-test yielded a t-value of approximately 0.27852 with 34 degrees of freedom, resulting in a *p*-value of approximately 0.7823. The high *p*-value suggested weak evidence against the null hypothesis, indicating no statistically significant difference between the means of the two groups at the 5% significance level. Additionally, the 95 percent confidence interval for the difference in means ranged from −5.597 to 7.375, encompassing zero. This further supported the conclusion that there was no significant difference in the MP concentration values between the vertical horizons.

Considering this analysis, we acknowledge that the differences observed in core 4 (showing higher MP concentrations in the top section) are not statistically significant. Future studies may want to consider analyzing a greater number of cores’ subsections to further explore the distribution.

Based on this result, it is suggested that the plastic particles exhibited a tendency to uniformly aggregate throughout the ice core. This is consistent with previous studies conducted on Arctic Sea ice, where plastic concentrations agreed with heterogeneous distribution [11,12,13]. However, having only had the opportunity to analyze three cores prohibited a definitive statistical outcome.

### 3.2. Microplastics in Chukchi and Beaufort Seawaters

As delineated in Section 2.3, no blank assessments were conducted to mitigate atmospheric contamination during the sampling and preparation processes on the SKQ0202014S cruise. Consequently, we will present quantitative data independently for fibers and all other microplastic particle categories.

**Figure 4:**
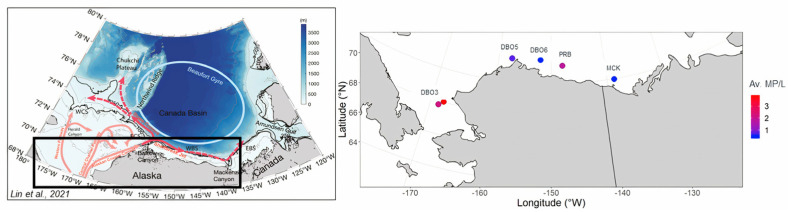
In the left panel, we present the schematic circulation of the western Arctic Ocean, adapted from Lin et al. (2021) [26]. The right panel displays a map of the study area, with dots representing microplastic concentrations, and a color code indicating the magnitude of microplastic concentrations (MP/L) along the Beaufort and Chukchi seawater. The data represent averaged values per site.

**Figure 5:**
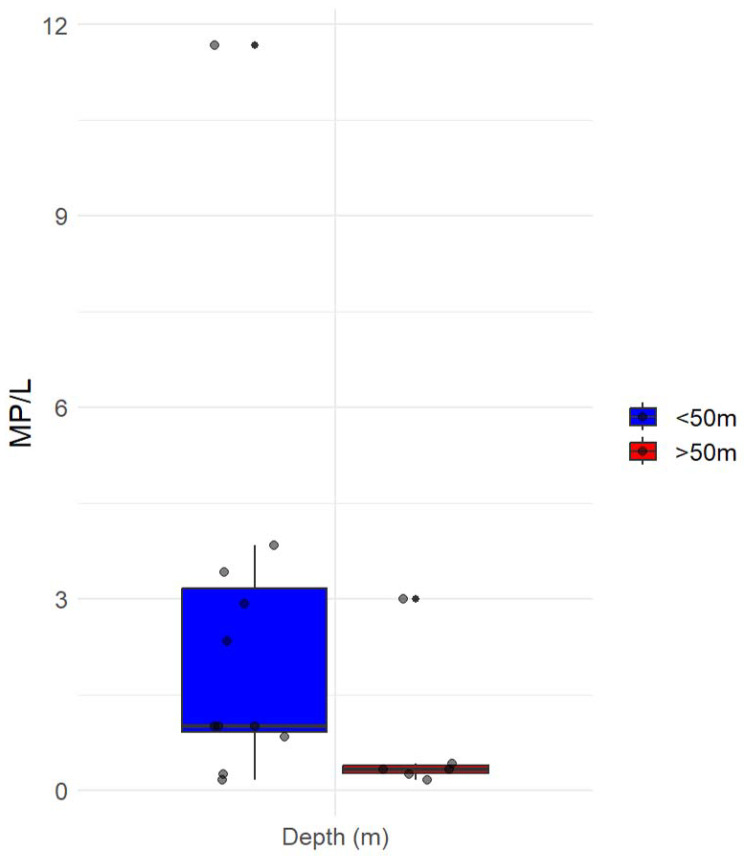
The boxplot with quartiles included represents the vertical distribution of the number of the median value of microplastic particles per liter of seawater greater and less than 50 m below the sea surface. Jittered points are overlaid on the box plots to visualize individual data points within each group. Any data points beyond the whiskers are considered potential outliers and are plotted individually.

Overall, the MP particles occurring in the seawater samples, excluding fibers, showed an average of 1.9 MP/L, with a maximum of 11.7 MP/L in surface waters. The MP per liter of seawater were greater at the Distributed Biological Observatory (DBO) stations where water was collected in the Chukchi Sea (Figure 4), and across the entire survey region in waters shallower than 50 m (Figure 5). The average concentration of plastic particles in the Chukchi Sea water samples was 3.6 ± 4 MP/L, while the Beaufort Sea samples recorded an average of 1 ± 1 MP/L. The difference in concentrations recorded was ~56% as the Chukchi Sea water was transported into the Beaufort Sea (Figure S5).

**Figure 6:**
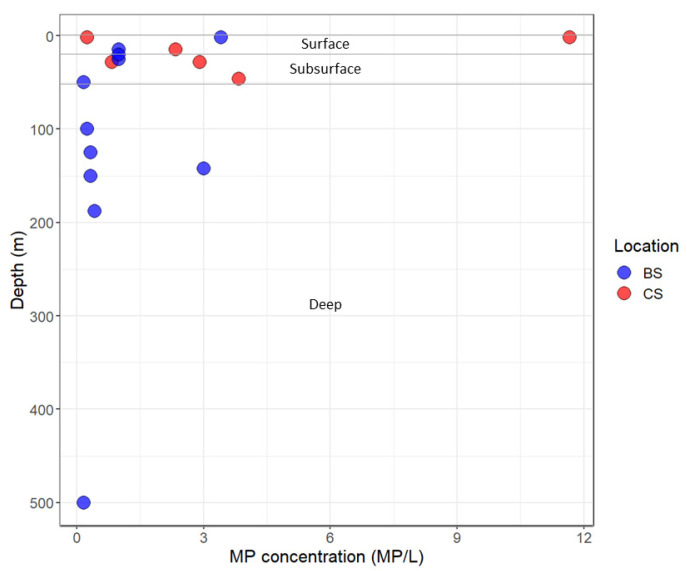
Scatter plot illustrating the vertical distribution of microplastic (MP) samples collected in seawater. The color code represents the sampling location, with blue indicating the Beaufort Sea (BS) and red representing the Chukchi Sea (CS).

Considering the different bathymetry of the two sites, we categorized the samples based on their depth into three distinct groups: surface samples (0–20 m), subsurface samples (20–50 m), and deeper samples (50 m to the bottom). Surface samples demonstrated an average microplastic concentration of 3.3 ± 4 MP/L, subsurface samples displayed an average concentration of 1.7 ± 1.5 MP/L, and deeper samples exhibited an average concentration of 0.75 ± 1 MP/L (Figure 6). These observations indicate a greater prevalence of microplastics in the shallow water column, likely influenced by the composition of microplastics and hydrodynamic processes specific to that region.

**Figure 7:**
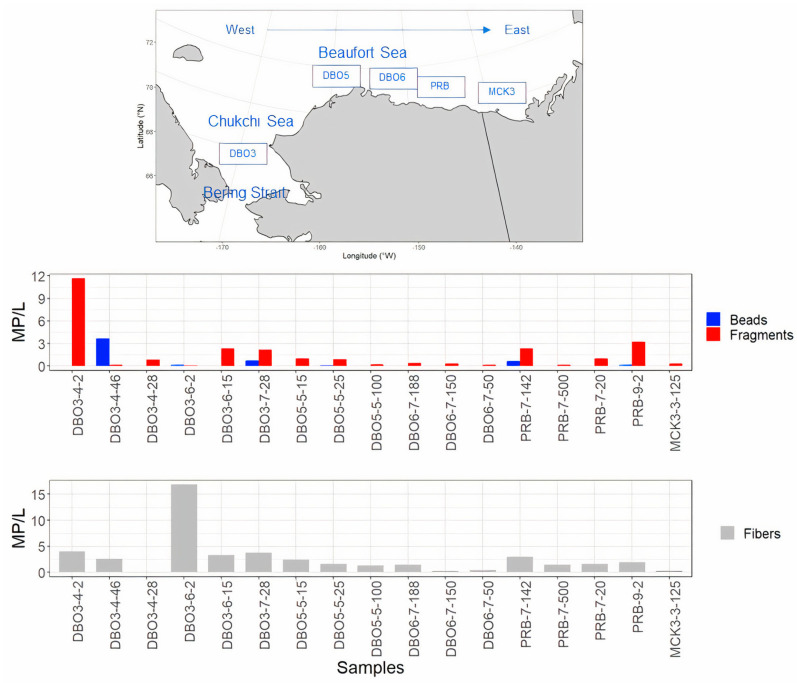
This figure illustrates microplastic concentrations per sample using color-coded bars to represent various shape types (fiber, fragment, bead). Additionally, a map provides an overview of the sample locations. The upper box displays data for bead and fiber particles, while the lower box (in grey) focuses on fibers. Numerical values accompanying the station ID indicate the depths at which the samples were collected.

Fibers and fragments were the predominant plastic shape types observed at all stations. Nevertheless, due to the absence of a blank control during sampling, these results may have been subject to bias. Additionally, beads particles were identified in 35% of the analyzed samples. Notably, the samples collected at station DBO3, located near the Bering Strait along the Alaskan Coastal Current, exhibited the highest concentrations of plastic particles for each shape type (Figure 7). Conversely, stations situated northeast of DBO3, progressing towards the Beaufort Sea, displayed lower counts of microplastic particles (refer to Figure 4 and Figure 7).

### 3.3. Microplastic Composition within Sea Ice and Seawater

**Figure 8:**
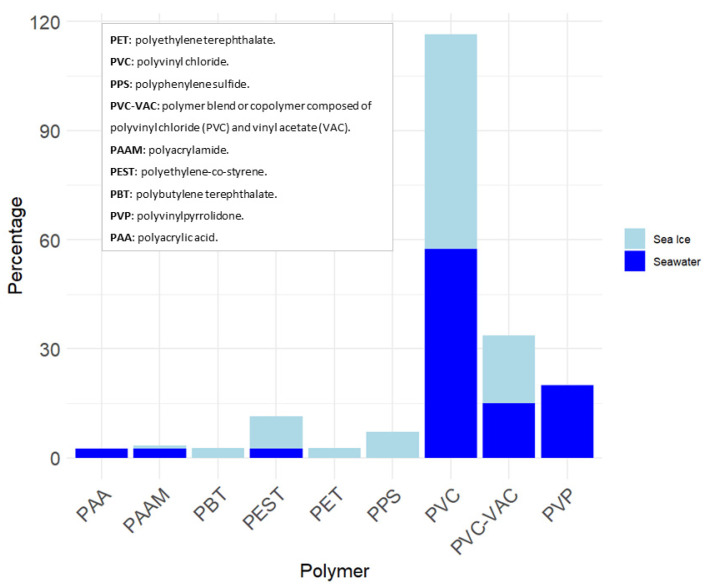
Polymer composition of sea ice and seawater samples. The percentages presented in this figure were calculated based on a dataset consisting of 84 samples for sea ice and 35 samples for seawater. The data shown represent samples with a siMPle score greater than 0.06.

Overall, polyvinyl chloride (PVC) emerged as the most prevalent polymer across the distinct compartments (Figure 8).

The polymer composition exhibited low variability across the different compartments analyzed. In the sea ice samples, a total of seven distinct polymer species were identified, while six polymers were detected within the seawater samples. However, the number of samples analyzed in seawater versus sea ice could have biased the results. Interestingly, the low-density polymers PAA and PVP were not found in sea ice samples, whereas high-density polymers, such as PPS, PET, and PBT were not detected in the seawater samples (Figure S6). This result indicates that high-density polymers likely underwent sedimentation and accumulated on the seafloor instead of remaining suspended in the water column. The findings were further supported by the analysis of seabed surface samples collected during the campaign, although the concentrations of MPs were not quantified, and the Raman spectroscopy data supporting these findings are presented in the supplementary material (Figure S7). Nonetheless, it is crucial to acknowledge that the density of polymers can be altered via weathering processes in the environment [49,50].

## 4. Discussion

### 4.1. MP in Sea Ice

The concentration of microplastics observed in the sea ice of the Canadian Arctic Archipelago (CAA) in the summer 2019 was found to be consistent with a more recent study conducted in the Central Arctic region [13].

The studies conducted by Kanhai et al. (2020) [13], Peeken et al. (2018) [12], and Obbard et al. (2014) [11] have documented variations in the composition of plastics found in sea ice. Specifically, fibrous polyesters, polyamides, and polyethylene were identified as the predominant types of plastics in their investigations. In contrast, our study primarily detected PVC in the sea ice samples. Similar to previous findings (e.g., [13]), our results lack a consistent pattern in the vertical distribution of microplastics within the sea ice cores. However, it is important to note that our study was limited to only three sea ice cores, which may have impacted the ability to observe consistent patterns.

The introduction of microplastics into our sea ice samples is hypothesized to originate from Pacific waters entering the Arctic Ocean through the Bering Strait, as suggested by Peeken et al. (2018) [12]. The Chukchi, Beaufort, and East Siberian Seas, which adjoin the central Arctic, are known to be influenced by Pacific waters [9]. Consequently, sea ice formed in these regions has the potential to contain microplastics present in the surface waters of these areas. This implies a potential pathway for the incorporation of microplastics into the sea ice, which can subsequently be released during the melting season.

To comprehensively understand the dynamics of microplastics within sea ice, it would be beneficial to gather microplastic data from multiple seasons when sea ice is formed. This would allow for a more comprehensive assessment of the quantity of microplastics that become frozen within first-year ice, as well as the amount released during the melting process.

### 4.2. MP in Seawater

Our study was able to provide more observations in the Beaufort Sea for microplastic particle counts focusing on the seasonal sampling period when historically, microplastics in seawater have been observed, as exemplified in [6]. It is of the utmost importance to consider the methodological variations when comparing and interpreting the findings from previous studies [6,9,10]. The distinct characteristics of different sampling methods, such as the neuston net, manta net, and Niskin bottles, can significantly impact the volume of water sampled. For example, the neuston net predominantly captures materials from the water surface, leading to a potential underestimation of the total water volume sampled. Conversely, the manta net allows for a more comprehensive representation of microplastics throughout the water column, owing to its larger sampling volume. The Niskin bottle facilitates controlled and discrete water sampling at specific depths, enabling researchers to target particular features and acquire samples with known water volumes.

**Figure 9:**
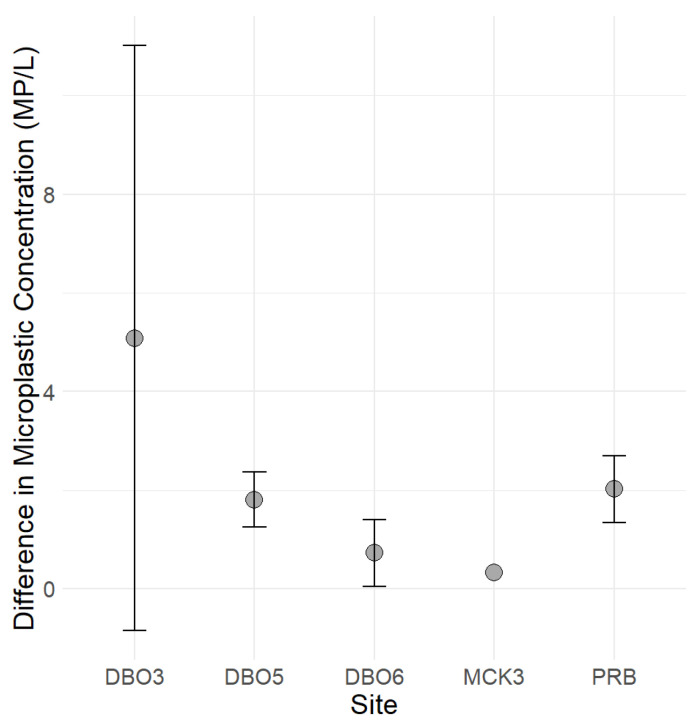
Differences in microplastic concentration (MP/L) between samples with and without fibers at different sites. Grey dots represent the mean percentage difference, while error bars denote one standard deviation above and below the mean, highlighting the impact of fibers on the overall concentration.

Given these variations in the sampling methods, it is crucial to express microplastic concentrations in appropriate units that account for the characteristics of the respective sampling tools. As a result, the studies cited in the present work have employed different units for microplastic concentration, reflecting the diversity in sampling approaches and their associated volumes. Consequently, a direct comparison of the magnitude of microplastic concentrations across these studies is not feasible. Furthermore, we were unable to perform blank control runs during the sampling procedure. Consequently, we acknowledge the potential for bias in the recorded fiber content within our samples. Nevertheless, when comparing data that includes fibers to data that excludes them, our analysis shows that the overall microplastic concentration remained spatially consistent. Notably, the presence of fibers in the samples, particularly in sample DBO3 (refer to Figure 9), resulted in a significantly higher magnitude. This outcome suggests that fibers had a substantial influence on the final result in DBO3, while overall, they had a limited impact on the total concentration of microplastics observed.

It is crucial to acknowledge that, in this study, we have categorized all fibers as external contaminants. However, we are aware that fibers constitute the most prevalent type of microplastics found in the environment (e.g., references [9,10,16,51]). Therefore, our approach can be considered an attempt to compensate for the absence of control measures for airborne contamination.

In the Chukchi and Beaufort Sea, microplastic concentrations in seawater may correspond to the degree of offshore sea ice melt and river runoff [18,31], but further studies are required to determine the microplastic source contributions to microplastic concentration [10]. As of now, contrasting results have emerged on MP in the Chukchi Sea. Ikenoue et al. (2023) [10] reported a wide range of particle and mass inventories of MP in the Chukchi Sea, which could be indicative of variability in their distribution. They compared the inventories in the Chukchi Sea with those in the Arctic Ocean on the Atlantic side and with the global ocean, suggesting lower pollution levels in the Chukchi Sea. This paper also highlighted that the annual flux of MP from the Pacific Ocean into the Chukchi Sea was significantly higher than the total amount of MP present in the Chukchi Sea water, indicating the accumulation of microplastics in other reservoirs. It was reported to have the lowest microplastic concentration in the Arctic Ocean (second to the Laptev Sea) at 5236 ± 6127 pieces km^−2^ [10]. On the other hand, Mu et al. (2019) [9] reported the abundance of MPs in surface waters across the Northwestern Pacific, the Bering Sea, and the Chukchi Sea. Mu et al. (2019) [9] identified the Chukchi Sea as having the highest level of MP among the studied regions. Our study results contradicted those of Ikenoue et al. (2023) [10] and corroborated the findings of Mu et al. (2019) [9], which underscores the importance of further tailored investigations in this region.

The Chukchi Sea and Beaufort Sea exhibit distinct bathymetric characteristics, which must be considered when analyzing samples in a vertical context. In the Chukchi Sea, the maximum water column depth was 57 m, while the deepest point in the Beaufort Sea reached 747 m.

Sample DBO5-5, located near Barrow Canyon, provided evidence of a decrease in MP concentrations in the area where the northward-flowing Alaskan Coastal Current bifurcates into the Chukchi Slope Current and Beaufort Shelf jet (refer to Figure 1). Previous studies have shown that water transport at Barrow Canyon, predominantly originating from the Pacific, is facilitated by this seabed feature [28,29,30]. It is possible that this feature also plays a role in the direction of microplastic transport into the Beaufort Basin, but a larger dataset is required to determine this. It is worth noting that no samples were collected at depths exceeding 50 m at site DBO3. Consequently, we can only speculate on how the vertical distribution of MP/L changes as water masses are transported northeastward and along the Beaufort Shelf.

These results underscored the importance of considering location-specific factors and local environmental conditions when studying microplastic pollution in marine ecosystems, as the variability in microplastic levels between different sites can have significant implications for understanding and addressing the impact of microplastics on marine life and ecosystems. Further investigations into the sources and distribution of microplastics in these regions are warranted to develop targeted mitigation strategies and conservation measures. However, it is important to note that there is currently no standardized method for processing microplastic samples. This lack of standardization makes it challenging to reliably compare and interpret the recorded data from different studies. Therefore, caution should be exercised when making direct comparisons between studies due to variations in sampling methods, sample processing techniques, and analytical protocols. Standardizing these methodologies in future research would enhance the comparability and reliability of microplastic data in the Arctic region. Further research and standardized methodologies are crucial for better comparability and assessment of microplastic pollution in the Arctic region.

## 5. Conclusions

This study provides evidence for the ubiquitous presence of microplastics in both sea ice cores and seawater in the western Arctic Ocean. The vertical distribution of microplastics revealed elevated concentrations primarily within the upper 50 m of the water column, while the most substantial accumulation was observed in the sea ice. Our findings supported the existing literature that has identified sea ice as a sink for microplastics [11,12,13]. On a spatial scale, we observed a higher distribution of microplastics in seawater in the Chukchi Sea, with a decreasing trend towards the east, while the CAA sea ice did not show a longitudinal trend in microplastic concentration. This distribution pattern in seawater could potentially be attributed to the dynamical influence of the Barrow Canyon. Our observations at Barrow Canyon suggest that water transport through this seabed feature acts as a major pathway for microplastic transport, directing microplastics deeper into the Beaufort Basin where shelf break processes may cause microplastics to sink either onto the seafloor or flow to the surface and become embedded within sea ice. However, further studies are needed to determine and potentially distinguish this.

Seven different types of synthetic polymers were identified in the sea ice cores of the Canadian Arctic Archipelago (CAA), while six types were found in the waters of the Chukchi and Beaufort Seas. Among these, fibers and fragments of polyvinyl chloride were the most abundant microplastic types, comprising approximately 60% of the microplastics found in both sea ice cores and seawater samples.

Overall, this study contributes to our understanding of microplastic distribution and composition in the western Arctic Ocean, highlighting the importance of considering both sea ice and seawater compartments. Subsequent research endeavors should focus on amalgamating the dataset by concurrently collecting samples from distinct sea ice, the seawater column, and sediment within identical locations and time frames.

## Data Availability

All the data are collected in the supplementary material, Tables S1–S3. The sea ice salinity data were published by the University of Illinois at Chicago at https://doi.pangaea.de/10.1594/PANGAEA.937543 (accessed on 1 May 2023).

## Supplementary Materials

The following supporting information can be downloaded at: https://www.mdpi.com/article/10.3390/toxics11090792/s1, Figure S1: Bar plots showing the mean microplastic concentration (MP/L) in different sea ice (a) and seawater (b) samples (x-axis), represented by the blue bars. The red error bars indicate the range of variability, represented by the standard deviation (SD) around the mean microplastic concentration for each sample. Figure S2: Example of one of the images of the filter from core one (filter three) with the larger orange boxes depicting the ×10 objective, and smaller red boxes depicting the ×50 objective where spectra were acquired. The blue crosses show the exact locations where spectra were acquired. Figure S3: Raman spectrum of a polyvinyl chloride (PVC) particle, obtained with 50× magnification, along with a corresponding scanned particle image (10× magnification). The y-axis represents the laser intensity, while the x-axis displays the wavenumber (cm−1). Figure S4: Visualization of the simple software interface. The sample spectrum is represented by the orange line, while the blue line corresponds to the reference spectrum from the library of reference spectra for plastics version 1 (Primpke et al., 2020). The open box in the center indicates the software’s correlation score. Figure S5: Box plot showing the distribution of microplastic concentrations (MP/L) across different locations (Beaufort Sea–BS and Chukchi Sea–CS). The boxes represent the interquartile range (IQR), with the median indicated by the horizontal line inside each box. Whiskers extend to the minimum and maximum values within 1.5 times the IQR. Outliers are plotted as individual points beyond the whiskers. Figure S6: Density of polymers relative to seawater. Please note that the density values may vary due to weathering effects on plastic in the environment. Figure S7: Polymer composition of surface seabed samples. The percentages presented in this figure were calculated based on a dataset consisting of 24 samples for seabed sediments. The data shown represent samples with a simple score greater than 0.06. Table S1: Concentrations of microplastics (MP/L) in sea ice cores collected during the Northwest Passage Project Cruise. Table S2: Concentrations of microplastics (MP/L) from seawater samples collected during the SKQ0202014S Cruise. Table S3: Under-ice seawater samples collected at sea ice core stations (1, 2, 3, 4-5). Table S4: Control samples used to evaluate laboratory contamination during the filtration of seawater and sea ice samples. References [11,12,52,53,54,55] are cited in the supplementary materials.
