# Supplementary material for: Microplastics Distribution within Western Arctic Seawater and Sea Ice
Source: Toxics. 2023 Sep 20;11(9):792. doi: 10.3390/toxics11090792 (PMC10534329; doi:10.3390/toxics11090792)
Supplement: Supplementary file 1 [file toxics-11-00792-s001.zip › toxics-2541400-supplementary.pdf]

Supplementary material

Table S1: Concentrations of microplastics (MP/L) in sea ice cores collected during the Northwest Passage Project Cruise. Cores 1 and 2 were treated as a single unit, while cores 3, 4, and 5 were divided into two sections (0–20 cm and 20 cm–bottom) to evaluate the vertical distribution of microplastic particles. The term "Top" refers to the uppermost 20 cm of the sea ice core, while "Rest" denotes the remaining section of the core from 20 cm to the bottom. The lengths of the sea ice cores were as follows (in sequential order): 225 cm, 80 cm, 180 cm, 94 cm, and 90 cm.

Core section	Latitude (ºN)	Longitude (ºW)	Fibers (MP/L)	Fragments (MP/L)	Beads (MP/L)
1	74.278	-98.406	120.67	0.29	1.55
2	73.913	-96.780	4.87	0.30	0.03
3rest	74.100	-94.276	2.15	0.06	0.05
3top	74.100	-94.276	1.08	0.03	0.09
4rest	73.352	-89.299	3.58	0.42	0.00
4top	73.352	-89.299	6.93	1.01	0.03
5rest	73.352	-90.299	14.67	2.44	3.11
5top	73.352	-90.299	16.03	1.43	2.06

Table S2: Concentrations of microplastics (MP/L) from seawater samples collected during the SKQ0202014S Cruise. The sample IDs are as follows: Distributed Biological Observatory – DBO, Mackenzie Sound – MCK, and Prudhoe Bay – PRB, and the number at the end describes the depth at which the samples were collected.

Sample ID	Site	Depth (m)	Latitude (ºN)	Longitude (ºW)	Fibers (MP/L)	Fragments (MP/L)	Beads (MP/L)
DBO3-4-2	DBO3	2	68.129	-167.506	4.00	11.67	0.00
DBO3-4-46	DBO3	46	68.129	-167.506	2.58	0.17	3.67
DBO3-4-28	DBO3	28	68.129	-167.506	0.00	0.83	0.00
DBO3-6-2	DBO3	2	67.782	-168.595	16.83	0.08	0.17
DBO3-6-15	DBO3	15	67.782	-168.595	3.33	2.33	0.00
DBO3-7-28	DBO3	28	67.895	-168.236	3.75	2.17	0.75
DBO5-5-15	DBO5	15	71.411	-157.484	2.42	1.00	0.00

DBO5-5-25	DBO5	25	71.411	-157.484	1.67	0.92	0.08
DBO5-5-100	DBO5	100	71.411	-157.484	1.33	0.25	0.00
DBO6-7-188	DBO6	188	71.424	-152.043	1.50	0.42	0.00
DBO6-7-150	DBO6	150	71.424	-152.043	0.25	0.33	0.00
DBO6-7-50	DBO6	50	71.424	-152.043	0.42	0.17	0.00
PRB-7-142	PRB	142	71.101	-147.890	3.00	2.33	0.67
PRB-7-500	PRB	500	71.101	-147.890	1.50	0.17	0.00
PRB-7-20	PRB	20	71.101	-147.890	1.67	1.00	0.00
PRB-9-2	PRB	2	71.021	-147.984	1.92	3.25	0.17
MCK3-3-125	MCK3	125	69.972	-138.695	0.33	0.33	0.00

Table S3: Under-ice seawater samples collected at sea ice core stations (1, 2, 3, 4-5). The locations of the sampling sites are indicated in Figure 1.

Sample ID	Fibers (MP/L)	Fragments (MP/L)	Beads (MP/L)
W1	2	2	0
W2	0	0	0
W3	1	1	0
W4	0	3	0

Table S4: Control samples used to evaluate laboratory contamination during the filtration of seawater and sea ice samples.

Sample ID	Fibers (n. particles)	Beads (n. particles)	Fragments (n. particles)
C1.seawater	2	0	1
C2.seawater	0	0	1
C3.seawater	1	0	0
C4.seawater	0	0	0
C1.seaice	1	0	1
C2.seaice	2	0	0

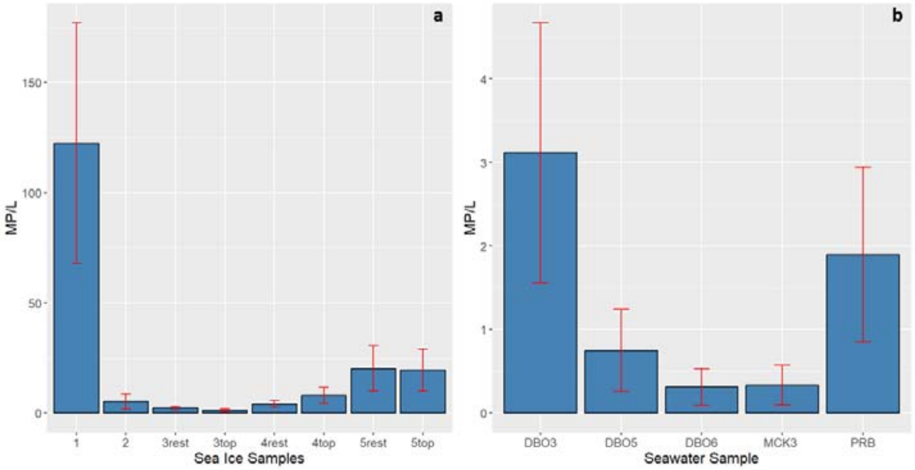

Figure S1: Bar plots showing the mean microplastic concentration (MP/L) in different sea ice (a) and seawater (b) samples (x-axis), represented by the blue bars. The red error bars indicate the range of variability, represented by the standard deviation (SD) around the mean microplastic concentration for each sample.

SOP S1: Standard Operating Procedures for Sampling and Processing Ice for Microplastics: A Method Developed Onboard the Icebreaker Oden during the Northwest Passage Project in July–August 2019.

Alessandra D'Angelo, Jacob Strock, and Brice Loose, University of Rhode Island (URI), Graduate School of Oceanography (GSO).

Procedure:

1. **Contamination Assessment:** a. Prepare pure controls using DI water to quantify contamination during sample collection and processing.

2. **Labware Preparation:** a. Thoroughly wash labware with DI water and ethanol to minimize contamination. b. Cover labware with tinfoil after cleaning and during use to prevent contamination. c. Use ethanol to reduce surface tension as recommended in previous studies (Nuelle et al., 2014; Obbard et al., 2014; Peeken et al., 2018).

3. **Fiber Contamination Prevention:** a. Wear non-fibrous waterproof jackets to prevent the contamination of fibers.

4. **Clean Bench Use:** a. Conduct the entire sample processing procedure in a clean bench to maintain a controlled environment and reduce contamination.
5. **Sample Collection and Transport:** a. Collect sea ice cores using the Kovacs core driller. b. Place the cores in transparent plastic bags for transport onboard.
6. **Sample Preparation:** a. Cut the sea ice cores using a steel saw. b. Rinse the core sections with DI water. c. Divide the core into two sections: 0–20 cm and 20 cm–bottom, and let them melt in foil bags.
7. **Filtration Process:** a. Filter the melted volume onto 0.7 µm Whatman GF/F and 0.2 µm Millipore Nitro Cellulose filters, as specified by Obbard et al. (2014). b. Process the remaining 20 ml through the FlowCam with a 300 µm flow cell and 10x objective.
8. **Sample Storage:** a. Store the filters in bumped foil paper inside sterile plastic bags. b. Freeze the filters at -20°C for post-cruise analysis.

Note: Throughout the procedure, be vigilant in preventing any potential contamination. Properly label and document each step and ensure that all labware and equipment are cleaned before use.

SOP S2: Standard Operating Procedures for analyzing Raman spectra for polymer identification: method implemented at URI Rhode Island Consortium of Nanoscience and Nanotechnology (RIN2).

Irene Andreu, Alessandra D’Angelo, and Laura Glastra. URI, RIN2, GSO.

Procedure:

1. **Preparation:** a. Set up the microscope and ensure it is functioning properly. b. Prepare the glass slide to place the GF/F filter on it.
2. **Filter Placement:** a. Place the GF/F filter on the glass slide. b. Visualize the filter under the microscope at ×10 objective to center it properly.
3. **Setting Coordinates:** a. Once the filter is visually centered, set both X and Y coordinates to zero in the WITec software used for sample analysis.
4. **Image Mapping:** a. Adjust the width and height of the image in the software based on the size of the filter (10 cm). b. Set the upper limit for the Z axis to the maximum limit of the microscope to avoid collisions during the mapping process. c. Initiate the image mapping process, ensuring that each spectrum collected from the filter is associated with specific coordinates (Fig. S2).
5. **Spectra Collection:** a. After the filter has been mapped, continue to collect individual spectra from the four quadrants on the filter. b. Set the laser source to 785 nm and 10 mW of power. c. Use an integration time of 1 sec and perform 300 accumulations.

6. **Focus Adjustment:** a. Before acquiring the spectrum, ensure each particle is individually selected and focused under the ×10 objective. b. Once focused, switch to the ×50 objective to acquire the spectrum.

Note: Throughout the procedure, handle the GF/F filter with care to avoid damage or contamination. Clean the equipment and dispose of the filters properly after completing the analysis. Record all relevant data, such as the coordinates of the spectra collected, and any observations made during the analysis.

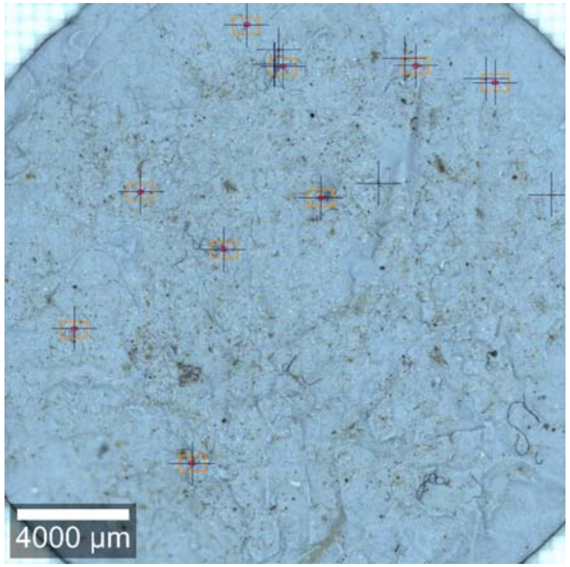

Figure S2: Example of one of the images of the filter from core one (filter three) with the larger orange boxes depicting the ×10 objective, and smaller red boxes depicting the ×50 objective where spectra were acquired. The blue crosses show the exact locations where spectra were acquired.

SOP S3: Standard Operating Procedures for processing Raman spectra for polymer identification: method implemented at URI Rhode Island Consortium of Nanoscience and Nanotechnology (RIN2).

Irene Andreu, Alessandra D'Angelo, and Laura Glastra, URI, RIN2, GSO.

The spectral processing was conducted using the WITec Five software version 5.2, employing the following steps for data processing:

1. **Cropping Data:**
    - Data outside the desired polymer range for plastics were cropped, specifically from wavenumber 200 cm^{-1}. This step focused the analysis on the relevant spectral region.
2. **Background Subtraction:**
    - Background noise was subtracted from the spectra using a shape–size range of Raman intensity (arb. Units) from 80 to 120. This process effectively removed unwanted background signals, enhancing the clarity of the polymer peaks.
3. **Cosmic Ray Subtraction:**
    - Any visible cosmic rays present in the spectra were subtracted. This step eliminated artifacts caused by cosmic rays, ensuring the reliability of the data.
4. **Spectral Smoothing:**
    - Spectral smoothing was implemented using the Savitzky–Golay algorithm. This technique contributed to noise reduction and improved the overall quality of the spectra.

It should be noted that during the processing step, there was a possibility that some minor peaks in the spectra might have been eliminated. However, it is important to acknowledge that previous studies have demonstrated the effectiveness of the employed data denoising methods, yielding satisfactory results (e.g., Yu et al., 2013; Kumar et al., 2014; Liu and Lunter, 2020).

By employing these data processing steps, the Raman spectra were effectively prepared for polymer identification, ensuring accurate and reliable data analysis, interpretation, and subsequent research findings.

References

Kumar, Nitendra, A. H. Siddiqi, and Khursheed Alam. "Raman spectral data de-noising based on wavelet analysis." International Journal of Computer Applications 102, no. 16, 2014.

Liu, Y, Lunter, DJ. Selective and sensitive spectral signals on confocal Raman spectroscopy for detection of ex vivo skin lipid properties. Translational Biophotonics; 2:e202000003. https://doi.org/10.1002/tbio.202000003, 2020.

Nuelle, M.T., Dekiff, J.H., Remy, D. and Fries, E. A new analytical approach for monitoring microplastics in marine sediments. Environmental pollution, 184, pp.161-169, 2014.

Obbard, R.W., Sadri, S., Wong, Y.Q., Khitun, A.A., Baker, I. and Thompson, R.C. Global warming releases microplastic legacy frozen in Arctic Sea ice. Earth's Future, 2(6), pp.315-320, 2014.

Peeken, I., Primpke, S., Beyer, B., Gütermann, J., Katlein, C., Krumpen, T., Bergmann, M., Hehemann, L. and Gerdts, G. Arctic sea ice is an important temporal sink and means of transport for microplastic. Nature communications, 9(1), pp.1-12, 2018.

Yu J, Wei W, Menyo MS, Masic A, Waite JH, Israelachvili JN. Adhesion of mussel foot protein-3 to TiO2 surfaces: the effect of pH. Biomacromolecules. Apr 8;14(4):1072-7. doi: 10.1021/bm301908y. Epub 2013 Mar 14. PMID: 23452271; PMCID: PMC3635841, 2013.

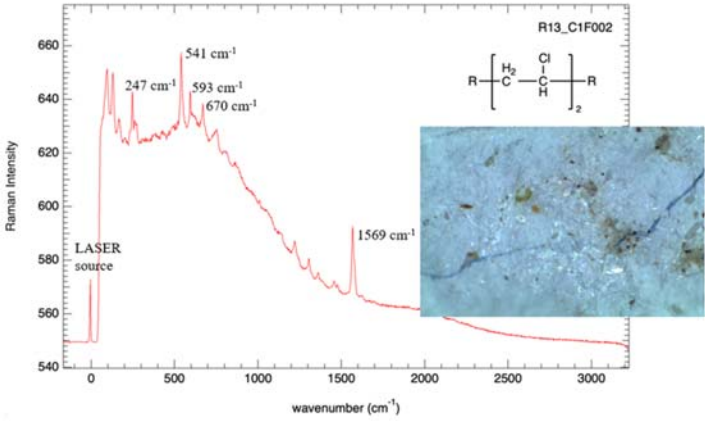

Figure S3: Raman spectrum of a polyvinyl chloride (PVC) particle, obtained with 50x magnification, along with a corresponding scanned particle image (10x magnification). The y-axis represents the laser intensity, while the x-axis displays the wavenumber (cm^{-1}).

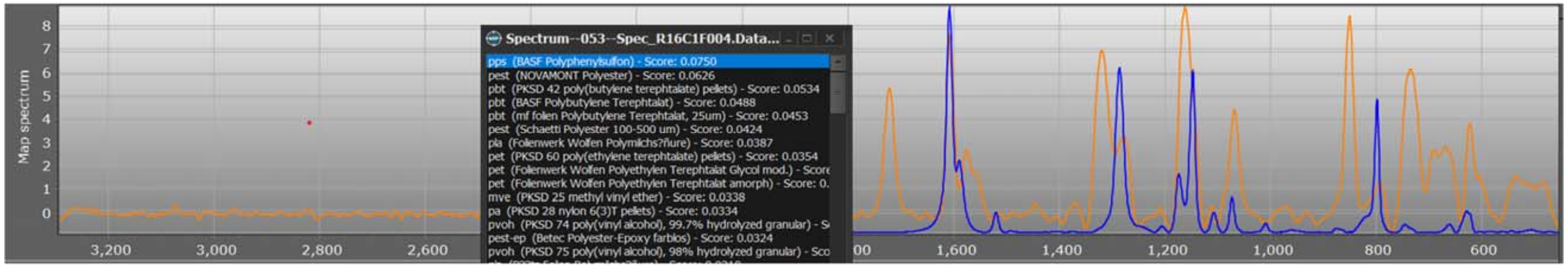

Figure S4: Visualization of the simple software interface. The sample spectrum is represented by the orange line, while the blue line corresponds to the reference spectrum from the library of reference spectra for plastics version 1 (Primpke et al., 2020). The open box in the center indicates the software's correlation score.

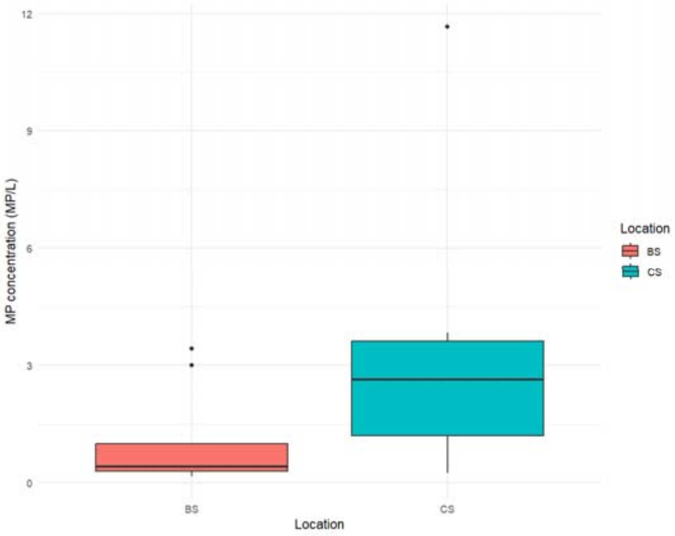

Figure S5: Box plot showing the distribution of microplastic concentrations (MP/L) across different locations (Beaufort Sea–BS and Chukchi Sea–CS). The boxes represent the interquartile range (IQR), with the median indicated by the horizontal line inside each box. Whiskers extend to the minimum and maximum values within 1.5 times the IQR. Outliers are plotted as individual points beyond the whiskers.

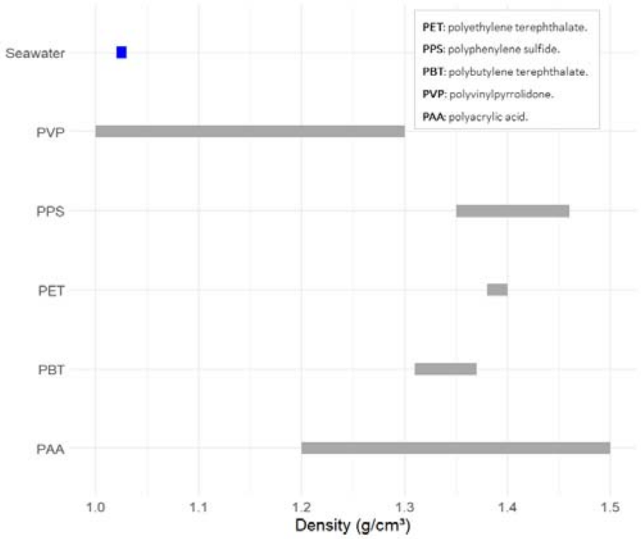

Figure S6: Density of polymers relative to seawater. Please note that the density values may vary due to weathering effects on plastic in the environment.

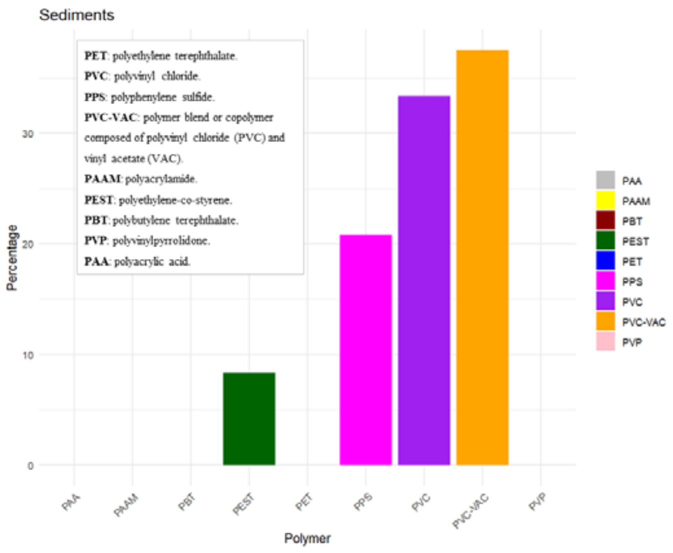

Figure S7: Polymer composition of surface seabed samples. The percentages presented in this figure were calculated based on a dataset consisting of 24 samples for seabed sediments. The data shown represent samples with a simple score greater than 0.06.
